# Characteristics of central nervous system progression in non‐small cell lung cancer treated with crizotinib or alectinib

**DOI:** 10.1002/cnr2.1414

**Published:** 2021-05-07

**Authors:** Hiroaki Sakamoto, Noriko Yanagitani, Ryo Manabe, Ryosuke Tsugitomi, Shinsuke Ogusu, Takehiro Tozuka, Hiroshi Yoshida, Yoshiaki Amino, Ryo Ariyasu, Ken Uchibori, Satoru Kitazono, Sadatomo Tasaka, Makoto Nishio

**Affiliations:** ^1^ Department of Thoracic Medical Oncology The Cancer Institute Hospital, Japanese Foundation for Cancer Research Tokyo Japan; ^2^ Department of Respiratory Medicine Hirosaki University Graduate School of Medicine Hirosaki Japan

**Keywords:** alectinib, CNS metastases, crizotinib, non‐small cell lung cancer

## Abstract

**Background:**

Most patients treated with anaplastic lymphoma kinase *(*ALK*)*‐tyrosine kinase inhibitors for *ALK*‐positive non‐small cell lung cancer (NSCLC) develop resistance, leading to metastasis, with progression to the central nervous system (CNS) being a primary concern. Although alectinib has better CNS penetration than crizotinib, patients treated with alectinib also develop CNS progression. CNS metastases more likely occurs during crizotinib treatment due to less blood‐brain barrier (BBB) penetration capability than alectinib. CNS progression pattern may be different during crizotinib and alecitinib treatment. Understanding the characteristics of CNS progression is important for developing treatment strategies.

**Aims:**

We compared the clinical‐radiographic characteristics of CNS metastases among patients undergoing crizotinib and alectinib treatment for *ALK*‐positive NSCLCs.

**Methods and results:**

We retrospectively analyzed the radiographic and clinical characteristics of CNS progression in *ALK*‐positive NSCLC patients treated with crizotinib or alectinib at our hospital between July 2011 and May 2020. CNS and systemic tumor progression were evaluated using computed tomography or magnetic resonance imaging. Fifty‐three and 65 patients were treated with crizotinib and alectinib, respectively. Baseline CNS metastasis was observed in 18 and 27 patients in the crizotinib and alectinib groups, respectively. Among the patients in the crizotinib and alectinib groups who developed disease progression, 15/49 (30.6%) and 9/44 (20.5%) had CNS progression, respectively (*P* = .344). Intra‐CNS progression‐free survival was significantly longer in the alectinib group than in the crizotinib group (median: 14.0 vs 5.6 months, *P* = .042). The number of CNS metastases sized ≥3 cm, rate of peritumoral brain edema, and the second progression pattern after treatment continuation was not significantly different between the groups.

**Conclusion:**

We observed no significant difference in the clinical‐radiographic characteristics of CNS progression between patients undergoing crizotinib and alectinib treatments. Local therapy, including stereotactic radiosurgery, for CNS progression may be suitable and important following alectinib and crizotinib treatment.

## 1 INTRODUCTION

Non‐small cell lung cancer (NSCLC), as the most predominant subtype of lung cancer, accounts for approximately 85%‐90% of all lung cancer cases.^1^ Many NSCLC cases are advanced at diagnosis and have a poor prognosis.^2^ However, the discovery of oncogenic driver mutations, such as epidermal growth factor receptor (*EGFR*) and anaplastic lymphoma kinase *(ALK)* rearrangement, has led to the development of molecular targeted therapy for NSCLC, which in turn has drastically improved patient survival.^3^, ^4^

In 2007, Soda et al identified NSCLC with *ALK* rearrangements, with 3%‐5% of NSCLC cases being *ALK* positive.^5^ Crizotinib is the first‐generation ALK‐tyrosine kinase inhibitor (TKI) that showed high efficacy for *ALK*‐positive NSCLC. Its phase III trials have demonstrated that crizotinib yielded significantly longer progression‐free survival (PFS) than cytotoxic chemotherapy.^4^, ^6^ After crizotinib, several ALK‐TKIs, such as alectinib, ceritinib, brigatinib, and lorlatinib that showed a long‐term survival benefit were approved for clinical use.^4^, ^7^, ^8^, ^9^, ^10^, ^11^, ^12^, ^13^, ^14^ ALK‐TKIs are currently a key drug for the treatment of *ALK*‐positive NSCLC.^15^, ^16^, ^17^ However, the majority of patients treated with ALK‐TKIs develop resistance, typically within 12 months, leading to disease progression.^18^, ^19^, ^20^ The central nervous system (CNS) is commonly the first site of progression, occurring in 46% of cases without systemic progression and 85% of *ALK*‐positive patients who were treated with crizotinib.^21^, ^22^

In phase III PLOFILE1014 study, crizotinib achieved a better rate of intracranial disease control compared with cytotoxic chemotherapy; however, there was no significant difference in the time to CNS progression between the two treatments.^23^ This may be partly attributed to the poor CNS penetration of crizotinib as a result of p‐glycoprotein‐mediated efflux through the blood‐brain barrier (BBB).^24^

As another ALK‐TKI, alectinib is a second‐generation highly selective and potent drug. Alectinib is not a substrate of p‐glycoprotein and has improved CNS penetration in contrast to crizotinib.^24^ J‐ALEX study conducted in Japanese patients^13^ and global ALEX study^7^ directly compared alectinib and crizotinib in patients with advanced *ALK*‐positive NSCLC, whereby alectinib achieved significantly better PFS. Moreover, alectinib showed better efficacy for CNS progression than crizotinib, in the subgroup analysis of the J‐ALEX study.^25^

Differences in the efficacy of ALK‐TKIs for CNS are considered to be due to differences in the capability to penetrate the BBB.^26^ Compared to crizotinib, second‐generation ALK‐TKIs, including alectinib have a better capability to penetrate the BBB. The BBB penetration capacity (%, cerebrospinal fluid [CSF]/blood) of crizotinib is only 0.26% (human model), whereas that for alectinib is 63%‐94% (animal model).^27^ The better BBB penetration capability of alectinib is considered one of the reasons for the lower incidence rates of CNS metastases and longer time to CNS progression with alectinib treatment.

However, despite alectinib treatment, a long‐term follow‐up found cumulative incidence rates of CNS progression at approximately 30% and 10% in patients with and without baseline CNS metastases, respectively.^28^

Therefore, treatment strategies after CNS progression are important in *ALK*‐positive NSCLC, even if alectinib becomes a standard treatment in first‐line treatment. Local therapy with either stereotactic radiosurgery (SRS) or whole‐brain radiotherapy for isolated CNS progression during *EGFR*‐ or ALK‐TKI treatment is recommended^29^ because in several reported cases, patients with *EGFR‐* and *ALK*‐positive NSCLC who developed isolated CNS progression achieved long PFS following local therapy with the maintenance of the same TKI therapy.^22^

Contrarily, leptomeningeal metastasis (LM) has also been reported after first‐generation *EGFR*‐TKIs in *EGFR*‐positive NSCLC, and resistance mutations such as T790M are known to be one of the causes of LM.^30^ If a patient developed systemic progression, multiple CNS metastases, or LM, local therapy for CNS progression would not be recommended.^29^

Furthermore, because CNS metastases cause impairment in the quality of life and performance status, the characteristics of CNS progression are also important.^31^, ^32^, ^33^

Crizotinib and alectinib have different BBB penetration capabilities; therefore, different CNS progression patterns are expected between patients undergoing the two treatments. Although there are some studies on the incidence of CNS progression in ALK‐TKIs, no study has compared the characteristics of CNS progression between patients undergoing crizotinib and alectinib treatments.

The American Society of Radiation Oncology guidelines on Radiotherapeutic and Surgical Management for Newly Diagnosed Brain Metastasis(es) stipulates that the number of CNS metastases (single or multiple), the maximum tumor size (less or more than 3‐4 cm), and expected prognosis are important decision‐making factors for radiosurgery, whole‐brain radiotherapy, surgery, and palliative care for CNS metastases.^34^ Therefore, the number of CNS metastases, the expected prognosis, and the maximum tumor size are important for determining the subsequent treatment for CNS progression. In addition, the LGK0901, a multi‐institutional prospective observational study of SRS for patients with brain metastases, showed that extracerebral disease status, neurological symptoms, and LM are also important factors.^35^

Because it may help in determining treatment strategies following CNS progression, this study aimed to investigate the radiographic and clinical characteristics of newly developed or progressed CNS metastases with crizotinib treatment compared to those with alectinib treatment for *ALK*‐positive NSCLC.

## 2 METHODS

### 2.1 Study design and patients

This was a retrospective study of patients with advanced *ALK*‐positive NSCLC treated with crizotinib or alectinib at the Cancer Institute Hospital of the Japanese Foundation for Cancer Research between July 2011 and May 2020. *ALK* positivity was determined either via immunohistochemistry and/or fluorescence in situ hybridization. Crizotinib was administered at a dose of 250 mg twice daily, while alectinib was administered at 300 mg twice daily. Patients who progressed or newly developed CNS metastases during the treatment period were included in the analysis.

### 2.2 Assessments

Tumor responses were assessed using the Response Evaluation Criteria in Solid Tumors version 1.1 (RECIST 1.1).^36^ CNS and systemic tumor progression were assessed using computed tomography (CT) or magnetic resonance imaging (MRI) of the chest, abdomen, and brain. Image scans for tumor assessment were performed at baseline, between 4 and 8 weeks, and then every 1 to 3 months until the treatment discontinuation. These scanned images were retrospectively reviewed by radiologists and thoracic oncologists.

The number of CNS metastases, the maximum size of CNS metastases, the number of CNS metastases measuring ≥3 cm in size, presence/absence of peritumoral brain edema, and presence/absence of LM were assessed as radiographic characteristics. Meanwhile, presence/absence of CNS symptoms, presence/absence of extra‐CNS progression, intra‐CNS PFS, local therapy for CNS progression, the number of patients who continued treatment after CNS progression, time to treatment failure(TTF) from CNS progression, and the site of progression after treatment continuation were assessed as clinical characteristics.

CNS symptoms were defined as headache, paralysis, nausea, and seizure in this study. We defined CNS progression as intracranial disease progression based on the RECIST 1.1. Intra‐CNS PFS was calculated from the start date of ALK‐TKIs until CNS progression. TTF from CNS progression was defined as the time from the first CNS progression to ALK‐TKIs discontinuation beyond the first CNS progression in patients who underwent local therapy after CNS progression and continued ALK‐TKIs beyond.

### 2.3 Statistical analysis

Between‐group comparisons of age and the size of brain metastases were conducted using the Mann‐Whitney *U* test. Meanwhile, Fisher's exact test or Pearson's chi‐square test compared the other patient characteristics as appropriate. Intra‐CNS PFS and time to treatment failure were evaluated using the Kaplan‐Meier method and compared between groups using the log‐rank test. All statistical analyses were performed using EZR (Saitama Medical Center, Jichi Medical University, Saitama, Japan) for statistical computing.^37^ *P* value of <0.05 was considered statistically significant.

## 3 RESULTS

### 3.1 Patient characteristics

**FIGURE 1:**
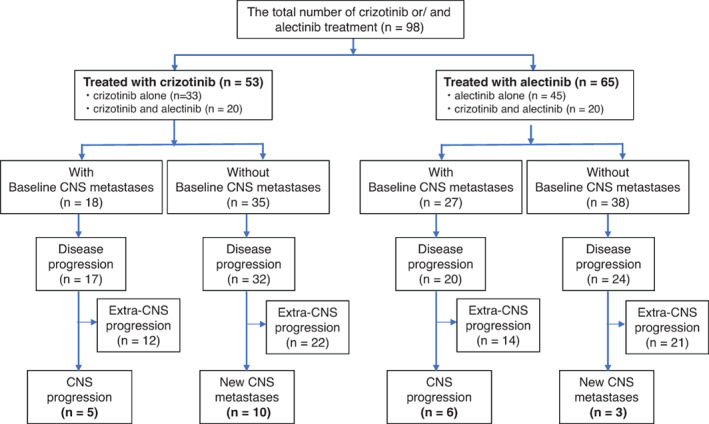
Patient inclusion flowchart. Thirty‐three patients received crizotinib alone, 45 patients received alectinib alone, and 20 patients received both crizotinib and alectinib. Disease progression or death occurred in 49/53 patients in the crizotinib group and 44/65 patients in the alectinib group

In total, 98 patients with *ALK‐*positive NSCLC received crizotinib alone (n = 33 patients) or alectinib alone (n = 45 patients). Of them, 20 patients were treated with both crizotinib and alectinib. The patient inclusion flowchart is shown in Figure 1. The median patient age in the crizotinib group was 48 years, 21 patients were male, and 27 were never smokers. Meanwhile, the median patient age in the alectinib group was 55 years, 34 patients were male, and 39 were never smokers. With respect to histology, a majority of the patients had adenocarcinoma, and the Eastern Cooperative Oncology Group performance status score was 0‐1 in 44 and 56 patients in the crizotinib and alectinib groups, respectively. There was no significant difference in baseline characteristics between the patients with CNS progression in the two groups. The patients' characteristics are shown in Table 1.

**TABLE 1:** Patient characteristics

		Total			CNS progression		
		Crizotinib	Alectinib	*P*‐value	Crizotinib	Alectinib	*P*‐value
		(n = 53)	(n = 65)		(n = 15)	(n = 9)	
Age	Median (range)	48 (22‐77)	55 (26‐83)	.027	44 (34‐76)	45 (33‐58)	.531
Sex	Male/female	21/32	34/31	.170	3/12	5/4	.100
Smoking history	Never/ex or current	27/26	39/26	.324	13/2	3/6	.635
Histology	Adeno/non‐adeno	52/1	64/1	.884	15/0	9/0	1.000
ALK test	IHC/FISH/both/unknown	11/15/24/3	25/12/26/2	.168	6/6/3/0	1/4/4/0	.253
PS	0,1/≥2	44/9	56/9	.638	11/4	5/4	.412
Baseline CNS metastases	With/without	18/35	27/38	.399	5/10	6/3	.206
Previous treatment							
Chemotherapy	None/1 prior chemotherapy/≥2 prior chemotherapy	13/24/16	40/15/10	.0003	6/4/5	5/2/2	.867
Prior ALK‐TKI	None/crizotinib/alectinib/other ALK‐TKIs/2 or more ALK‐TKIs	44/‐/6/2/1	44/18/‐/0/3	–	11/‐/1/0	4/3/‐/2	–

Abbreviations: ALK, anaplastic lymphoma kinase; CNS, central nervous system; FISH, fluorescence in situ hybridization; IHC, immunohistochemistry; PS, performance status; TKI, tyrosine kinase inhibitor.

CNS metastases at baseline were observed in 18 and 27 patients in the crizotinib and alectinib groups, respectively. Among the 53 patients in the crizotinib group, 40 patients had received prior chemotherapy, 44 patients had not received prior ALK‐TKIs, 6 patients had received prior alectinib, 2 patients had received prior other ALK‐TKIs, and 1 patient had received 2 or more prior ALK‐TKIs. Among the 65 patients in the alectinib group, 25 patients had received prior chemotherapy, 44 patients had not received prior ALK‐TKIs, 18 patients had received prior crizotinib, and 3 patients received 2 or more ALK‐TKIs. Disease progression was observed in 49 and 44 patients in the crizotinib and alectinib groups, respectively. Of them, 15/49 (30.6%) patients in the crizotinib group and 9/44 (20.5%) patients in the alectinib group developed CNS progression (*P* = .344). None of the patients progressed to CNS metastases in either group. Overall, 11/44 (25.0%) and 4/44 (9.1%) patients without prior ALK‐TKIs in the crizotinib and alectinib groups, respectively, developed CNS progression (*P* = .087). There were 5/18 (27.8%) and 6/27 (22.2%) patients with baseline metastases who developed CNS progression in the crizotinib and alectinib groups, respectively (*P* = .732). Meanwhile, 10/35 (28.6%) and 3/38 (7.9%) patients who had no baseline metastases developed CNS progression (*P* = .031).

### 3.2 Radiographic characteristics

The radiographic characteristics of CNS metastases were evaluated in 24 patients with CNS progression. With respect to modality, CNS metastases were assessed via CT in 23 patients (95.8%) and via MRI in 1 patient (4.2%). The radiographic characteristics classified by groups are shown in Table 2. Single CNS metastasis was observed in 7/15 (46.7%) and 2/9 (22.2%) patients in the crizotinib and alectinib groups, respectively. Meanwhile, multiple CNS metastases (ie, ≥5 metastases) were observed in 4/15 (26.6%) and 3/9 (33.3%) patients in the crizotinib and alectinib groups, respectively. The median maximum tumor size of CNS metastases was 9.2 mm (range, 5.0‐20.4 mm) and 9.1 mm (range, 2.0‐17.9 mm) in the crizotinib and alectinib groups, respectively (*P* = .682). No CNS metastases sized ≥3 cm in size were observed in both groups. Peritumoral brain edema was observed in 3/15 (20.0%) and 4/9 (44.4%) patients in the crizotinib and alectinib groups, respectively (*P* = .356). LM was observed in 1/15 (6.7%) and 2/9 (22.2%) patients in the crizotinib and alectinib groups, respectively (*P* = .533).

**TABLE 2:** Radiographic characteristics of CNS metastases

CNS Metastases		Crizotinib Group	Alectinib Group	*P‐*value
		(n = 15)	(n = 9)	
Number of metastases	1/2‐4/≥5	7/4/4	2/4/3	.572
	1/≥2	7/8	2/7	.389
	1‐4/≥5	11/4	6/3	1.000
Maximum tumor size, mm	Median (range)	9.2 (5.0‐20.4)	9.1 (2.0‐17.9)	.682
Number of tumor measuring ≥3 cm	≥3 cm/<3 cm	0/15	0/9	1.000
Peritumoral brain edema	Yes/no	3/12	4/5	.356
Leptomeningeal metastasis	Yes/no	1/14	2/7	.533

Abbreviation: CNS, central nervous system.

### 3.3 Clinical characteristics

The patients' clinical characteristics are shown in Table 3. In total, 8 of the 24 (33.3%) patients had CNS‐related symptoms during CNS progression. CNS progression without extra‐CNS progression was observed in 9/15 (60.0%) and 6/9 (66.7%) patients in the crizotinib and alectinib groups, respectively (*P* = 1.000). The intra‐CNS PFS was significantly longer in the alectinib group than that in the crizotinib group (median: 14.0 months [range, 1.8‐49.9 months] vs 5.6 months [range, 0.8‐33.9 months], *P* = .042). Overall, 3/15 (20.0%) and 4/9 (44.4%) patients in the crizotinib and alectinib groups underwent radiosurgery for CNS metastases, respectively. In addition, there were 4/15 (26.7%) patients who continued crizotinib and 4/9 (44.4%) patients who continued alectinib treatment after CNS progression (*P* = .412).

**TABLE 3:** Clinical characteristics of CNS metastases

		Crizotinib Group	Alectinib Group	
Clinical Characteristics		(n = 15)	(n = 9)	*P‐*value
Symptom with CNS metastases	With/without	5/10	3/6	1.000
Extra‐CNS progression	With/without	6/9	3/6	1.000
Intra‐CNS PFS, months	Median (range)	5.6 (0.8‐33.9)	14.0 (1.8‐49.9)	.042
Treatment for CNS metastases	Radiosurgery or surgery/WBRT	3/1	4/0	1.000
Treatment continuation after CNS progression	Yes/no	4/11	4/5	.412
Time to treatment failure after CNS progression, months	Median (range)	23.8 (1.7‐50.1)	15.5 (3.6‐18.1)	.652
Site of progression after treatment continuation	CNS/extra‐CNS/CNS and extra‐CNS	0/3/1	2/1/0	.257

Abbreviations: CNS, central nervous system; PFS, progression‐free survival; WBRT, whole‐brain radiotherapy.

**FIGURE 2:**
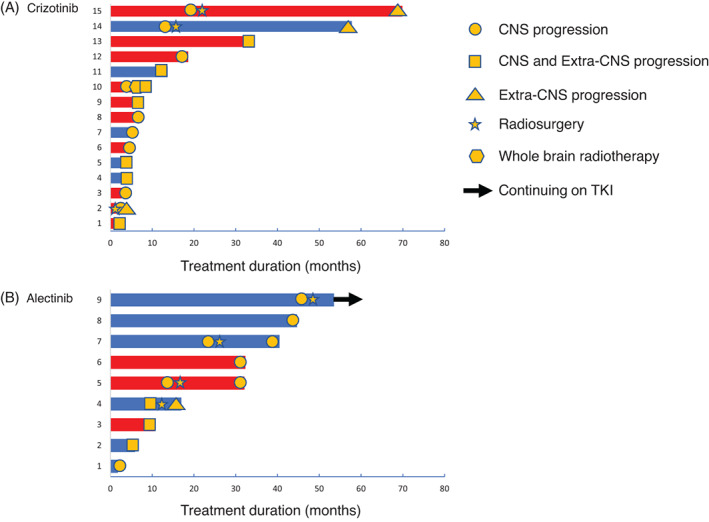
Treatment duration in the crizotinib group (A) and the alectinib group (B). The red and blue bars indicate with and without baseline CNS metastases, respectively. Patient 4 in the alectinib group developed progression to malignant pleural effusion; the patient underwent pleurodesis and continued alectinib treatment

The treatment time course is shown in Figure 2. The time to treatment failure after CNS progression was longer in the crizotinib group than in the alectinib group, but the difference was not significant (median: 23.8 months vs 15.5 months, *P* = .652).

Similarly, we did not observe a significant difference in the second progression pattern after the continuation of crizotinib and alectinib. Extra‐CNS progression after CNS progression was observed in four patients who continued crizotinib, and it was observed in one patient who continued alectinib (*P* = .257).

## 4 DISCUSSION

Few studies have focused on the characteristics of CNS progression in *ALK*‐positive NSCLCs. In the current study, although the incidence rates of CNS progression tended to be higher in those treated with crizotinib than in those treated with alectinib, the difference was not significant. However, the incidence rates of CNS progression in patients without baseline CNS metastasis were significantly lower in the alectinib group than those in the crizotinib group. Intra‐CNS PFS was also significantly longer in the alectinib group. These results are in line with those of previous reports^25^, ^38^ and confirm the superiority of alectinib to crizotinib for cases of *ALK*‐positive NSCLC.

In the phase III J‐ALEX study, the 1‐year cumulative incidence rates of CNS progression were 16.8% and 5.9% in the crizotinib and alectinib arms, respectively.^25^ The better BBB penetration capability of alectinib is considered one of the reasons for the lower incidence rates of CNS metastases and a longer time to CNS progression observed with alectinib treatment. Accordingly, we speculated that the difference in the BBB penetration capability might lead to differences in the characteristics of CNS progression between patients treated with crizotinib and alectinib. However, we did not observe a significant difference in the radiographic characteristics of CNS progression between the two treatment groups. CNS progression without extra‐CNS progression was observed in 9/15 (60.0%) and 6/9 (66.7%) patients in the crizotinib and alectinib groups, respectively. Among patients with CNS progression, those with progression in more than five sites, with metastases measuring <3 cm, and without LM, which is an indication of radiosurgery, was slightly lower in the alectinib group (44.4% vs 53.3%); however, this difference was also not significant. Similarly, the clinical characteristics observed after CNS progression had no significant difference.

In total, 3/15 (20.0%) patients in the crizotinib group and 4/9 (44.4%) patients in the alectinib group underwent radiosurgery following CNS progression.

In this study, there was no significant difference in the radiographic and clinical characteristics of CNS progression between patients undergoing crizotinib and alectinib treatments. Similar trends in patients with isolated CNS progression were observed in both treatment groups. Continuation of the drug beyond CNS progression may lead to long second PFS. This indicates that radiation therapy, including SRS for CNS metastases, may be as important as treatment strategies for CNS metastases during alectinib as CNS metastases during crizotinib.

This study has some limitations owing to its single‐institutional retrospective design and small sample size. The treatment varied among the patients, and therefore, we could not detect statistically significant differences owing to the small sample size. However, *ALK*‐positive NSCLC is relatively rare, and data on CNS progression after ALK‐TKIs are also limited. Thus, it is difficult to evaluate the characteristics of CNS progression in a large‐scale study. Despite these limitations, our study included a detailed description of the characteristics of CNS progression and, therefore, may be useful for establishing the optimal treatment strategy for *ALK*‐positive NSCLCs.

There was no significant difference in the radiographic and clinical characteristics of CNS progression between patients undergoing crizotinib and alectinib treatments. Local therapy, including SRS for CNS progression, may be suitable and important, following both alectinib or crizotinib treatment.

## CONFLICT OF INTEREST

Makoto Nishio reports honoraria from Ono Pharmaceutical, Bristol‐Myers Squibb, Pfizer, Chugai Pharmaceutical, Eli Lilly, Taiho Pharmaceutical, AstraZeneca, Boehringer‐Ingelheim, MSD, and Novartis and research funding from Novartis, Daiichi Sankyo, Taiho Pharmaceutical, Bristol‐Myers Squibb, Boehringer‐Ingelheim, Ono Pharmaceutical, Eli Lilly, Chugai Pharmaceutical, AstraZeneca, Merck Sernon, MSD, and Pfizer. Noriko Yanagitani reports an employment/leadership position/advisory role in Chugai Pharmaceutical. Ken Uchibori reports an employment/leadership position/advisory role in Daiichi Sankyo. All other authors have stated that they have no conflicts of interest.

## AUTHOR CONTRIBUTIONS

**Hiroaki Sakamoto:** Conceptualization; data curation; formal analysis; investigation; methodology; resources; visualization; writing‐original draft. **Ryo Manabe:** Data curation; resources. **Ryosuke Tsugitomi:** Data curation; resources. **Shinsuke Ogusu:** Data curation; resources. **Hiroshi Yoshida:** Data curation; resources. **Takehiro Tozuka:** Data curation; resources. **Yoshiaki Amino:** Data curation; resources. **Ryo Ariyasu:** Data curation; resources. **Ken Uchibori:** Data curation; resources. **Satoru Kitazono:** Data curation; resources. **Noriko Yanagitani:** Conceptualization; data curation; methodology; resources; writing‐review & editing. **Sadatomo Tasaka:** Writing‐review & editing. **Makoto Nishio:** Conceptualization; methodology; project administration; supervision; writing‐review & editing.

## ETHICAL STATEMENT

This study was approved by the Ethics Committee of the Cancer Institute Hospital of the Japanese Foundation for Cancer Research (approval number 2020‐1081) and was conducted according to the Declaration of Helsinki's guidelines. The need for informed consent was waived owing to the retrospective nature of the study.

## Data Availability

The data are available on reasonable request. The datasets used and/or analyzed during this study are available from the corresponding author on reasonable request.

## References

[1] Cataldo V , Gibbons D , Perez‐Soler R , Quintas‐Cardama A . Treatment of non‐small‐cell lung cancer with erlotinib or gefitinib. N Engl J Med. 2011;364(10):947‐955. 10.1056/NEJMct0807960. 21388312

[2] Morgensztern D , Ng S , Gao F , Govindan R . Trends in stage distribution for patients with non‐small cell lung cancer a national cancer database survey. J Thorac Oncol. 2010;5(1):29‐33. 10.1097/JTO.0b013e3181c5920c. 19952801

[3] Maemondo M , Inoue A , Kobayashi K , et al. Gefitinib or chemotherapy for non‐small‐cell lung cancer with mutated EGFR. N Engl J Med. 2010;365(25):2380‐2388. 10.1056/NEJMoa0909530. 20573926

[4] Solomon BJ , Mok T , Kim DW , et al. First‐line crizotinib versus chemotherapy in ALK‐positive lung cancer. N Engl J Med. 2014;371(23):2167‐2177. 10.1056/NEJMoa1408440. 25470694

[5] Soda M , Choi YL , Enomoto M , et al. Identification of the transforming EML4‐ALK fusion gene in non‐small‐cell lung cancer. Nature. 2007;448(7153):561‐566. 10.1038/nature05945. 17625570

[6] Shaw AT , Kim DW , Nakagawa K , et al. Crizotinib versus chemotherapy in advanced ALK‐positive lung cancer. N Engl J Med. 2013;368(25):2385‐2394. 10.1056/NEJMoa1214886. 23724913

[7] Peters S , Camidge DR , Shaw AT , et al. Alectinib versus Crizotinib in untreated ALK‐positive non‐small‐cell lung cancer. N Engl J Med. 2017;377(9):829‐838. 10.1056/NEJMoa1704795. 28586279

[8] Soria J‐C , Tan DSW , Chiari R , et al. First‐line ceritinib versus platinum‐based chemotherapy in advanced ALK ‐rearranged non‐small‐cell lung cancer (ASCEND‐4): a randomised, open‐label, phase 3 study. Lancet. 2017;389(10072):917‐929. 10.1016/S0140-6736(17)30123-X. 28126333

[9] Solomon BJ , Besse B , Bauer TM , et al. Lorlatinib in patients with ALK‐positive non‐small‐cell lung cancer: results from a global phase 2 study. Lancet Oncol. 2018;19(12):1654‐1667. 10.1016/S1470-2045(18)30649-1. 30413378

[10] Solomon B , Kim D , Wu Y , et al. Final overall survival analysis from a study comparing first‐line crizotinib versus chemotherapy in ALK‐mutation‐positive non‐small‐cell lung cancer. J Clin Oncol. 2018;36(22):2251‐2258. 10.1200/JCO.2017.77.4794. 29768118

[11] Rangachari D , Le X , Shea M , et al. Cases of ALK‐rearranged lung cancer with 5‐year progression‐free survival with crizotinib as initial precision therapy. J Thorac Oncol. 2017;12(11):e175‐e177. 10.1016/j.jtho.2017.06.002. 28611010 PMC5659921

[12] Watanabe S , Hayashi H , Okamoto K , et al. Progression‐free and overall survival of patients with ALK rearrangement‐positive non‐small cell lung cancer treated sequentially with crizotinib and alectinib. Clin Lung Cancer. 2016;17(6):528‐534. 10.1016/j.cllc.2016.05.001. 27318655

[13] Hida T , Nokihara H , Kondo M , et al. Alectinib versus crizotinib in patients with ALK ‐positive non‐small‐cell lung cancer (J‐ALEX): an open‐label, randomised phase 3 trial. Lancet. 2017;390(10089):29‐39. 10.1016/S0140-6736(17)30565-2. 28501140

[14] Camidge DR , Kim HR , Ahn MJ , et al. Brigatinib versus crizotinib in ALK‐positive non‐small‐cell lung cancer. N Engl J Med. 2018;379(21):2027‐2039. 10.1056/NEJMoa1810171. 30280657

[15] Rikova K , Guo A , Zeng Q , et al. Global survey of phosphotyrosine signaling identifies oncogenic kinases in lung cancer. Cell. 2007;131(6):1190‐1203. 10.1016/j.cell.2007.11.025. 18083107

[16] Koivunen JP , Mermel C , Zejnullahu K , et al. EML4‐ALK fusion gene and efficacy of an ALK kinase inhibitor in lung cancer. Clin Cancer Res. 2008;14(13):4275‐4283. 10.1158/1078-0432. 18594010 PMC3025451

[17] Takeuchi K , Soda M , Togashi Y , et al. RET, ROS1 and ALK fusions in lung cancer. Nat Med. 2012;18(3):378‐381. 10.1038/nm.2658. 22327623

[18] Katayama R , Shaw AT , Khan TM , et al. Mechanisms of acquired crizotinib resistance in ALK‐rearranged lung cancers. Sci Transl Med. 2012;4(120):120ra17. 10.1126/scitranslmed.3003316. PMC3385512 22277784

[19] Crystal AS , Shaw AT , Sequist LV , et al. Patient‐derived models of acquired resistance can identify effective drug combinations for cancer. Science. 2014;346(6216):1480‐1486. 10.1126/science.1254721. 25394791 PMC4388482

[20] Nishio M , Murakami H , Horiike A , et al. Phase I study of ceritinib (LDK378) in Japanese patients with advanced, anaplastic lymphoma kinase‐rearranged non‐small‐cell lung cancer or other tumors. J Thorac Oncol. 2015;10(7):1058‐1066. 10.1097/JTO.0000000000000566. 26020125 PMC4467585

[21] Toyokawa G , Seto T , Takenoyama M , Ichinose Y . Insights into brain metastasis in patients with ALK+ lung cancer: is the brain truly a sanctuary? Cancer Metastasis Rev. 2015;34(4):797‐805. 10.1007/s10555-015-9592-y. 26342831 PMC4661196

[22] Weickhardt AJ , Scheier B , Burke JM , et al. Local ablative therapy of oligoprogressive disease prolongs disease control by tyrosine kinase inhibitors in oncogene‐addicted non‐small‐cell lung cancer. J Thorac Oncol. 2012;7(12):1807‐1814. 10.1097/JTO.0b013e3182745948. 23154552 PMC3506112

[23] Solomon BJ , Cappuzzo F , Felip E , et al. Intracranial efficacy of crizotinib versus chemotherapy in patients with advanced ALK‐positive non‐small‐cell lung cancer: results from PROFILE 1014. J Clin Oncol. 2016;34(24):2858‐2865. 10.1200/JCO.2015.63.5888. 27022118

[24] Tang SC , Nguyen LN , Sparidans RW , Wagenaar E , Beijnen JH , Schinkel AH . Increased oral availability and brain accumulation of the ALK inhibitor crizotinib by coadministration of the P‐glycoprotein (ABCB1) and breast cancer resistance protein (ABCG2) inhibitor elacridar. Int J Cancer. 2014;134(6):1484‐1494. 10.1002/ijc.28475. 24037730

[25] Nishio M , Nakagawa K , Mitsudomi T , et al. Analysis of central nervous system efficacy in the J‐ALEX study of alectinib versus crizotinib in ALK‐positive non‐small‐cell lung cancer. Lung Cancer. 2018;121:37‐40. 10.1016/j.lungcan.2018.04.015. 29858024

[26] Okimoto T , Tsubata Y , Hotta T , et al. A low crizotinib concentration in the cerebrospinal fluid causes ineffective treatment of anaplastic lymphoma kinase‐positive non‐small cell lung cancer with carcinomatous meningitis. Intern Med. 2019;58(5):703‐705. 10.2169/internalmedicine.1072-18. 30333394 PMC6443566

[27] Nishino M , Soejima K , Mitsudomi T . Brain metastases in oncogene‐driven non‐small cell lung cancer. Transl Lung Cancer Res. 2019;8(Suppl 3):S298‐S307. 10.21037/tlcr.2019.05.15. 31857953 PMC6894990

[28] Nakagawa K , Hida T , Nokihara H , et al. Final progression‐free survival results from the J‐ALEX study of alectinib versus crizotinib in ALK‐positive non‐small‐cell lung cancer. Lung Cancer. 2020;139:195‐199. 10.1016/j.lungcan.2019.11.025. 31812890

[29] Camidge DR , Pao W , Sequist LV . Acquired resistance to TKIs in solid tumours: learning from lung cancer. Nat Rev Clin Oncol. 2014;11(8):473‐481. 10.1038/nrclinonc.2014.104. 24981256

[30] Wu YL , Zhao Q , Deng L , et al. Leptomeningeal metastasis after effective first‐generation EGFR TKI treatment of advanced non‐small cell lung cancer. Lung Cancer. 2019;127:1‐5. 10.1016/j.lungcan.2018.11.022. 30642536

[31] Kocher M , Soffietti R , Abacioglu U , et al. Adjuvant whole‐brain radiotherapy versus observation after radiosurgery or surgical resection of one to three cerebral metastases: results of the EORTC 22952‐26001 study. J Clin Oncol. 2011;29(2):134‐141. 10.1200/JCO.2010.30.1655. 21041710 PMC3058272

[32] Nayak L , Lee EQ , Wen PY . Epidemiology of brain metastases. Curr Oncol Rep. 2012;14(1):48‐54. 10.1007/s11912-011-0203-y. 22012633

[33] Peters S , Bexelius C , Munk V , Leighl N . The impact of brain metastasis on quality of life, resource utilization and survival in patients with non‐small‐cell lung cancer. Cancer Treat Rev. 2016;45:139‐162. 10.1016/j.ctrv.2016.03.009. 27019457

[34] Tsao MN , Rades D , Wirth A , et al. Radiotherapeutic and surgical management for newly diagnosed brain metastasis(es): an American Society for Radiation Oncology evidence‐based guideline. Pract Radiat Oncol. 2012;2(3):210‐225. 10.1016/j.prro.2011.12.004. 25925626 PMC3808749

[35] Yamamoto M , Serizawa T , Shuto T , et al. Stereotactic radiosurgery for patients with multiple brain metastases (JLGK0901): a multi‐institutional prospective observational study. Lancet Oncol. 2014;15(4):387‐395. 10.1016/S1470-2045(14)70221-9. 24621620

[36] Eisenhauer EA , Therasse P , Bogaerts J , et al. New response evaluation criteria in solid tumours: revised RECIST guideline (version 1.1). Eur J Cancer. 2009;45(2):228‐247. 10.1016/j.ejca.2008.10.026. 19097774

[37] Kanda Y . Investigation of the freely available easy‐to‐use software 'EZR' for medical statistics. Bone Marrow Transplant. 2013;48(3):452‐458. 10.1038/bmt.2012.244. 23208313 PMC3590441

[38] Gadgeel S , Peters S , Mok T , et al. Alectinib versus crizotinib in treatment‐naive anaplastic lymphoma kinase‐positive (ALK+) non‐small‐cell lung cancer: CNS efficacy results from the ALEX study. Ann Oncol. 2018;29(11):2214‐2222. 10.1093/annonc/mdy405. 30215676 PMC6290889

